# Person‐centred caregiver singing for people living with dementia in South Africa: A mixed methods evaluation of acceptability, feasibility, and professional caregivers' experiences

**DOI:** 10.1111/hex.13915

**Published:** 2023-11-17

**Authors:** Karyn Stuart‐Röhm, Imogen N. Clark, Felicity A. Baker

**Affiliations:** ^1^ The University of Melbourne Melbourne Victoria Australia; ^2^ Norwegian Academy of Music Oslo Norway

**Keywords:** acceptability, dementia, music therapy, person‐centred care, professional caregivers, singing, training

## Abstract

**Background:**

Dementia care in South Africa faces challenges including a paucity of published research, a prevalent medical model in healthcare, and inadequate caregiver training. Music is a meaningful psychosocial intervention in dementia care, yet its application is not always safe and effective. A training protocol was codesigned to enhance caregivers' delivery of person‐centred care through attuned, live singing.

**Objectives:**

This study explored the acceptability and caregivers' experiences of a person‐centred caregiver singing (PCCS) protocol in South Africa.

**Methods:**

A PCCS workshop was applied at seven aged care homes in Cape Town, South Africa. Forty‐one formal caregivers adhered to inclusion criteria and consented to attend one workshop on PCCS. Mixed methods data collection was obtained from questionnaires containing a Likert scale and written reflections. Quantitative data were analysed through nonparametric tests and narrative descriptions, and qualitative data through thematic content analysis. Findings were integrated deductively using seven components of acceptability.

**Results:**

Findings converged to show caregivers' positive experiences, highlighting observed improvements in residents' wellbeing, caregivers' capabilities, empathic connection, and person‐centred care beyond the one‐on‐one. Caregivers' limited song repertoire and residents' unpredictability hindered implementation, however, the skills acquired appeared useful and applicable.

**Conclusions:**

Integration of findings suggests the acceptability of PCCS as caregivers experienced PCCS as a helpful, easy‐to‐implement intervention that contributes to their delivery of person‐centred care. Further research focused on caregiver self‐efficacy, empathy, and caregivers' own personhood is needed as well as determining the most effective strategies to ensure maximum uptake and sustainability in the sector.

**Patient or Public Contribution:**

Formal caregivers participated in this study, both attending the training and implementing the singing protocol with residents in their care at their respective care homes. The people living with dementia residing at the care home were recipients of the singing protocol but not included as participants in the research.

## 1 INTRODUCTION

A dementia diagnosis presents economic, social, psychological, and physical challenges for those living with it, their family, caregivers, and society.^1^ Sixty percent of the approximate 50 million persons living with dementia reside in low‐ and middle‐income countries.^2^ In South Africa, estimates place the number of people living with dementia at 4% (187,000), although actual numbers may be closer to 11%.^3^ South Africa faces several challenges in the care of older persons, including a paucity of published dementia research and a lack of robust data,^3^, ^4^ and poor awareness of dementia amongst healthcare professionals and the public.^5^, ^6^ The lack of public funding, infrastructure, and limited services for older persons has led to formal caregiver (herein caregivers) training offered by private and nonprofit sectors.^3^ This training, which averages 3 months, is inconsistent in quality and duration and is not geriatric focused.^5^, ^7^ Additionally, the biomedical model remains prevalent in South Africa^8^ which limits access to person‐centred care.

Person‐centred care is regarded as an essential component for high‐quality long‐term provision of care for older people.^9^ This approach emphasises wellbeing and quality of life of people with dementia by recognising their individuality^10^, ^11^ and meeting their social and emotional needs.^12^ Dementia is a progressive disease characterised by a decline in a person's cognition affecting memory, intellect, comprehension, language, and judgement causing distressed behaviours^2^, hindering their ability to communicate physical and emotional needs^13^ and reducing the quality of life.^14^ Meeting these needs requires good communication skills, empathy, and a person‐centred approach.^15^, ^16^, ^17^ However, ineffective communication commonly predicates distressed behaviours and resistiveness.^18^ Meaningful communication is enhanced by recognition of personhood and attunement to the person with dementia,^15^ however such skills are not always included in standard caregiver training.^19^

Music is a common, cost‐effective, and easily implemented psychosocial intervention that facilitates communication and wellbeing of those with dementia.^20^, ^21^, ^22^, ^23^ Live singing by formal caregivers benefits the emotional wellbeing of the resident,^24^ eases the burden of care^25^ and enhances their relationship.^26^ However, its implementation and outcomes are not always consistent, safe, informed, or based on best practices.^27^, ^28^, ^29^ The safe use of music should be incorporated into formal caregivers' education as it benefits caregivers and people with dementia,^30^, ^31^ helps caregivers' respond and adapt better to residents' needs, and enhances the mutuality and reciprocity of their relationship.^32^ Although music therapists can play a vital role in such training,^31^, ^33^ there are few studies that include direct input from music therapists.^32^

Person‐centred caregiver singing (PCCS) is a caregiver‐delivered live music intervention model that seeks to support formal caregivers in their provision of person‐centred care to people living with dementia.^34^ It is defined as singing in a manner that employs various prosodic and empathic musical elements (the rhythm, tone, volume, tempo, intonation, emotion reflected in voice and language) to support communication and promote feelings of connection, safety, and validation to enhance the delivery of person‐centred care.^34^ The PCCS workshop is facilitated by a music therapist and aims to build caregivers' knowledge and insights into dementia, person‐centred care, affordances of music, and attunement using basic music therapy skills. Results from an action research study showed that PCCS was a helpful resource that eased care and created a positive caring environment by enhancing caregivers' capabilities, residents' emotional wellbeing, and the closeness in their relationship through effective communication.^34^

Our study built on the existing PCCS findings and examined the acceptability of the PCCS protocol with a larger cohort of formal caregivers across several care homes in South Africa. Our aim was to advocate and support the implementation of PCCS in care homes in South Africa and more broadly by addressing the following questions:

1. What are caregivers' experiences of the PCCS training?
2. What is the acceptability of the training and protocol by formal caregivers of people with dementia in care homes in South Africa?

## 2 MATERIALS AND METHODS

### 2.1 Design

We maintained a pragmatic stance^35^, ^36^ and employed mixed methods to answer the research questions. We developed a 20‐item questionnaire inf ormed by the theoretical frameworks and our previous work.^34^ Quantitative data were collected using ratings from a 5‐point Likert scale from 1 (*strongly disagree*) to 5 (*strongly agree*). Items focused on three aspects: (i) residents' observed responses, (ii) the caregiver's experiences and (iii) general impression of PCCS. Within each of these, space was provided for caregivers to document their own reflections in detail to allow for the collection of qualitative data. Supporting Information S1: Appendix A depicts the questionnaire. The length and literacy level of the questionnaire ensured reduced demand for caregivers' time and effort.^37^, ^38^ Two negatively phrased items were included to decrease potential response‐set bias.^39^ Reflexivity was maintained through regular discussions between authors.^40^

### 2.2 Participants

Author 1, who is a South African music therapist, approached several Cape Town care homes with dedicated dementia units (public and private). Seven care homes gave permission for interested caregivers to participate. After oral and written information was presented, caregivers volunteered and gave written informed consent to (1) attend a PCCS workshop facilitated by Author 1, a qualified music therapist, (2) implement PCCS during daily care of residents with dementia for 2 weeks and (3) complete a Likert questionnaire with the option to do so anonymously to reduce social pressure and bias.^41^

Forty‐one formal caregivers across seven care homes consented to participate. Caregivers were eligible to participate if they had received at least 3 months of professional caregiver training and were fluent in English. Although many caregivers expressed interest in participating, challenges with work conflicts arose. Forty‐one caregivers volunteered, but two did not meet the inclusion criteria. Table 1. presents the participant demographic information. In recognition of the importance of addressing organisational change and support of caregivers,^42^ we invited all staff members to attend the PCCS workshops. Several nursing managers, cleaning staff, occupational therapists, and human resources managers attended but were not enroled in the study and their data was not collected.

**Table 1:** Participant details.

	Formal caregivers (*n* = 39)
Ages	20–66 years (*M* = 41.51; SD = 11.66)
Gender	Female: *n* = 36, Male: *n* = 3
Culture Groups	Coloured: *n* = 28, Shona: *n* = 1, Zulu: *n* = 1, South Sotho: *n* = 1, isiXhosa: *n* = 8
First language	Afrikaans: *n* = 24, Shona: *n* = 1, isiXhosa: *n* = 7, English: *n* = 5, Sesotho: *n* = 1
Caregivers training duration (months)	3–12 months (*M* = 4.8; SD = 2.71)
Dementia caregiving experience (years)	0.5–22 years (*M* = 8.02; SD = 5.55)

### 2.3 Care homes settings

Seven care homes with dedicated dementia/frail care wards consented to participate in this study. Five care homes were categorised as under‐resourced nonprofit and/or charitable organisations reliant on private funding (*n* = 1) or subsidised by the government (*n* = 4). Of these, four were in low‐socioeconomic suburbs (as identified through income, levels of unemployment, and service/amenities provision) and one in a more affluent suburb. Two care homes were categorised as private, located in affluent areas, and provided access to specialised multidisciplinary services. Although familiar with these suburbs, Author 1 was not familiar with these particular care homes, and none of the care homes had any prior experience with music therapy services. It is noteworthy that the majority of residents of the care homes were white and Afrikaans/English speakers, and the majority of the caregivers were non‐White and Xhosa/Afrikaans speakers.

### 2.4 PCCS intervention

PCCS training aimed to share knowledge and build skills in sensitive, attuned live singing, within a person‐centred approach. This training was initially designed by Author 1 and was corefined alongside formal caregivers in a previous action research study.^34^ Training provided information on affordances of music and singing for self and people with dementia (e.g., mood regulation)^24^; the physiological, emotional, and social impact of music; dementia; personhood^11^; the VIPS framework^43^; and musical elements which refers to the building blocks of music, including dynamics, harmony, melody, rhythm, texture, timbre and tonality.^44^ The VIPS framework encapsulates person‐centered care principles into four elements: Valuing the person with dementia, Individualised care; Personal perspectives, and creating a positive Social environment.^43^ The VIPS framework encapsulates person‐centered care principles into four elements: Valuing the person with dementia, Individualised care; Personal perspectives, and creating a positive Social environment.^43^ Two basic music therapy skills of matching (playing or singing in a way that matches or fits in with the client's style of playing while maintaining the same tempo, dynamic, texture, and quality of their musical elements) and mirroring (doing what the client is doing musically, expressively and through body language at the same time) were included.^45^ The training included experiential components including music‐making, singing, and role‐playing scenarios using PCCS. Posttraining, caregivers were able to sing in a way that is responsive to the person with dementia, their symptoms, and behaviours at the moment; apply music therapy skills; and sing in a manner that adapts to these elements during daily care routines.

### 2.5 Data collection

Over a period of 4 months, nine training workshops were held onsite during working hours and facilitated by Author 1. Two weeks later, caregivers completed the Likert Scale Questionnaire. Thirty‐seven completed questionnaires were collected. Quantitative data were inputted into Excel and qualitative responses into MAXQDA 2022.^46^

### 2.6 Data analysis

The quantitative data from the Likert scale were analysed using Excel software. We employed nonparametric tests to measure frequencies and percentages of responses and described these using a narrative. Negative items (Q15, Q19) were reverse‐coded.^39^ We planned to use Cronbach's *α* to test for internal consistency.

A six‐step thematic content analysis^47^ employed for the qualitative data involved: (1) familiarisation with data; (2) inductive generation of initial codes^48^; (3) codes grouped into potential descriptive themes, (4) revision of themes and codes, (5) writing definitions, and naming them (using quotes), and (6) writing findings up. Verification occurred consistently through reference to original data and discussions between all authors.

Qualitative and quantitative findings were integrated deductively according to affective attitude, burden, perceived effectiveness, ethicality, intervention coherence, opportunity costs, and self‐efficacy.^49^

## 3 RESULTS

Thirty‐seven questionnaires were completed. One participant was lost to illness, and another participant left their employment at the care home. Internal consistency of the scale using Cronbach's *α* was high (*α* = .9) suggested strong similarity between items.^50^, ^51^

### 3.1 Quantitative data analysis

**Figure 1:**
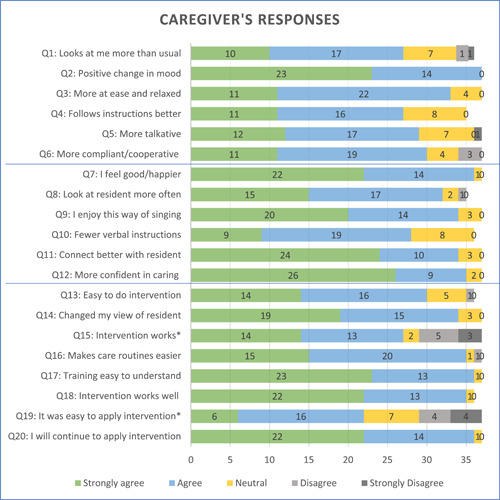
Bar graph depicting caregivers' responses. *Item reversed.

Once negative statements were reversed (Q15, Q19), results indicated a positive clustering towards agree and strongly agree in all categories (see Figure 1 for response frequencies). In the category *Resident's Observed Responses* (Q1–Q6), all 37 caregivers observed a positive change in residents' mood (Q2), which received the highest number of ‘strongly agreed’ responses. Although most caregivers (*n* = 27) agreed that residents followed verbal instructions (Q4), this yielded the most ‘neutral’ (*n* = 8) and nonresponses (*n* = 2). In *the Caregiver's Experiences* category (Q7–Q12), items relating to caregivers' confidence generated the highest number of ‘strongly agree’ responses (*n* = 26). Q10 relating to instructions received the most neutral responses (*n* = 8). In *General Impression of the Intervention* (Q13–Q20), caregivers strongly agreed with several items (Q17, Q19, Q20). The most ‘disagree’ and ‘strongly disagree’ responses were noted in both reversed questions relating to the intervention works (Q15) and its ease of application (Q19).

**Figure 2:**
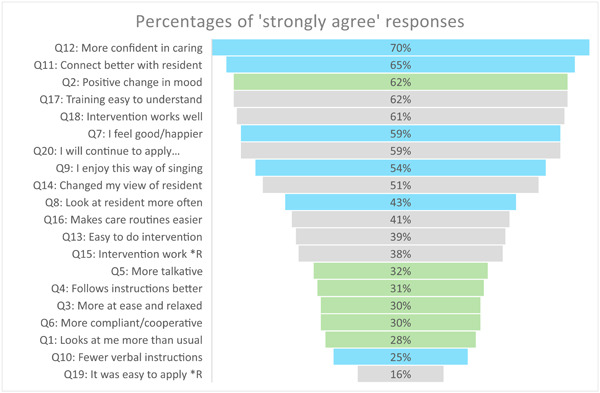
Graph depicting percentages of ‘strongly agree’ responses in order from highest to lowest.

Comparisons between categories indicated a greater number of ‘strongly agree’ responses in *Caregivers' Experiences* and more neutral responses in *Residents' Observed Responses*. This is confirmed in Figure 2 where most ‘strongly agree’ responses for *Caregivers' Experiences* (indicated in blue) clustered above those relating to *Residents' Observed Responses* (indicated in green). Responses in grey relate to *General Impression of the Intervention*.

### 3.2 Qualitative thematic analysis

After collating the caregivers' written comments into an Excel spreadsheet, Author 1 familiarised themselves with these, generated and grouped codes into descriptive and analytic themes. These were revised in discussion with Authors 2 and 3. Table 2 depicts an example of the analysis process.

**Table 2:** Example of analysis process.

Excerpt	Code	Descriptive theme	Analytic theme
When we are singing a song we know they love, they light up and try to sing along (CG_3). They are cheerful and smile more (CG_27).	Residents' recognition and awareness. Improved mood. Cheerfulness. Emotional expression.	Residents' awareness. Residents' shift in mood.	*PCCS promotes resident's psychosocial wellbeing*.
My day feels lighter, and tasks goes with joy and ease (CG_14). Made me feel more excited, confident and it's a very nice new way, but definitely works more better for me (CG_21).	Caregiver lighter mood. Easier caregiving. Caregiver more confident. PCCS motivated caregiver.	Caregiver improved emotional wellbeing. Caregiver improved self‐efficacy.	*PCCS supports caregivers' emotional capabilities*.

Abbreviation: PCCS, person‐centred caregiver singing.

Analysis revealed four analytic themes: Residents' psychosocial wellbeing; Caregivers' emotional capabilities; Caring between and caring beyond; and Challenges to PCCS' implementation.

### 3.3 Theme 1: Resident's psychosocial wellbeing

#### 3.3.1 Shifts in mood

Caregivers observed the residents' positive responses during PCCS. Singing seemed to enliven the residents, yet also calmed and relaxed them, especially when singing residents' favourite songs. Caregivers noted how this helped ease caregiving.

They are cheerful and smile more. (CG_27)

Resident change to a more relaxed mood which makes it easier for the carer to help resident. (CG_1)

The resident's mood is more uplifted and calmer, sleeps better at night. (CG_21)

#### 3.3.2 Active engagement in care

Residents appeared more alert, attentive and participated actively in their own care tasks. PCCS eased care by enhancing the resident's verbal and nonverbal communication, flexibility, and compliance.

I noticed they give you more attention when you sing with them. (CG_33)

Resident even help when you have to wash or dress them. Resident move arms and legs more to make it easy to dress. (CG_34)

She was never talkative, but when singing to her, she looked around and said ‘lovely’ with a broad smile. (CG_25)

### 3.4 Theme 2: PCCS supports caregivers' emotional capabilities

#### 3.4.1 Emotional wellbeing

Caregivers reported feeling happier, more content, and at ease with residents. They described feeling energised and strengthened, thereby reducing their burden of care. Caregivers depicted the PCCS workshop as a positive, helpful, and enjoyable experience. They appreciated the insights into dementia and affordances of music and hoped for more workshops.

My day feels lighter, and tasks goes with joy and ease. (CG_14)

It gives me more strength and energy. (CG_27)

I feel less stressed and just feel like doing it all day, I'm happy and more at ease to see the residents happy make me feel happy. (CG_32)

#### 3.4.2 Self‐efficacy

PCCS helped the caregivers feel confident and better able to support the residents. They described feelings of accomplishment, motivation, and perseverance. Additionally, some caregivers seemed to adopt a more flexible approach to caregiving, even when the resident did not appear interested in the singing.

As music is part of our daily lives, it seems easy to just make it work in our daily routines to help ease people with dementia's life. I'm looking forward to singing with them more. (CG_4)

Made me feel more excited, confident and it's a very nice new way, but definitely works more better for me. (CG_21)

Yes, some of them don't care for music or don't like it, some like the quietness and then I'll play birds singing and then she would smile, without saying a word. (CG_25)

### 3.5 Theme 3: Caring between and caring beyond

#### 3.5.1 Empathy through connection

Caregivers conveyed a better understanding of residents, recognising and adapting to their individual preferences by adapting their musical elements. The enhanced communication and connection was described as aiding smoother, easier care routines and a positive shift in the care atmosphere.

Because sometimes I can relate to how they are feeling. (CG_15)

The volume and speed I communicate to the patient make them feel more calmer. (CG_12)

Singing connects you and resident. Listen, observe the movement or the action comes from the resident while you sing and act on it. Be alert of the rhythm, pace also important. (CG_5)

There's better communication between the carer and the resident. There's always a smile on their face when they sing. (CG_29)

#### 3.5.2 Beyond the one‐on‐one

The application of PCCS extended beyond the one‐on‐one care routine. Some caregivers commented on its' helpfulness in group settings and in motivating residents to walk. Many advocated for other care homes and caregivers to attend PCCS workshops.

I tried the singing in group. Works wonders. Residents that were just passively sitting, mood changes, everybody looks alive, smiling and looks happier. I will keep doing this and using this method. (CG_14)

… when struggling to walk with the resident, when singing, they either start to dance or walk to the beat of the song. (CG_18)

### 3.6 Theme 4: Challenges to PCCS' implementation

Some caregivers mentioned that residents did not always respond to their singing. This was attributed to a resident's mood or their dislike of music. Caregivers also reported they did not always know songs familiar to residents. One caregiver found it challenging to adapt the routine when the resident resisted participation.

Not all of them feel the same. Some feel frustrated depends on which tone of the song you sing. And some feel calm. Depends also on what time you do the singing. (CG_13)

Sometimes the songs that they know or want to sing, the words we as the ‘new generation’ don't know. (CG_18)

It's difficult to get the resident out of that ‘distant’ or ‘leave me alone’ type of mood, it feels odd leaving the resident who doesn't want to participate and going on to the next person. (CG_18)

### 3.7 Integration of qualitative and quantitative findings

We integrated the quantitative and qualitative findings relating to the seven components of acceptability: affective attitude, burden, perceived effectiveness, ethicality, intervention coherence, opportunity costs, self‐efficacy.^49^ Six of the seven components were evident in the integrated data, with opportunity costs not emerging.

Findings demonstrated caregivers' positive attitudes towards PCCS in daily care. Caregivers appeared more confident (Q12, Theme 2) as it was easy to apply (Q13, Q19), worked well with residents (Q18, Theme 1, 3), made caring easier (Q16, Theme 2), and was a mutually enjoyable experience (Q2, Q3, Q7, Q9, Themes 1, 2, 3). The impact on caregivers' own mood (Q7), enjoyment (Q9), and confidence (Q12) aligned with their enhanced emotional well‐being and self‐efficacy (Theme 2). Self‐efficacy was further illustrated by increased accomplishment and motivation (Q6, Q20, Theme 2). Intervention coherence was demonstrated by caregivers' insight and understanding of PCCS confirmed by their confidence in application (Q12, Q13, Q17, Theme 2), their shift in their view of dementia (Q14, Theme 3), the benefits observed in residents (Q1–Q6, Theme 1) and their mutual and reciprocal connections (Q11, Theme 3). Their shift in attitudes, more empathic understanding and value of communication and connection (Q1, Q5, Q8, Q10, Q11, Q14, Theme 3) may also imply the presence of ethicality and how PCCS fits with the caregivers' value system. Findings highlighted benefits to nonverbal and verbal communication (Q1, Q5, Q8, Theme 1) and most caregivers agreed residents were more compliant (Q6, Q20, Themes 1, 3). Caregivers' responses reflected low burden associated with workshop participation, cognitive effort, and PCCS application (Q16, Theme 2) and they would continue using it (Q20, Theme 2, 3). Potential challenges impacting the burden and efficacy of PCCS included challenges of residents' unpredictable moods, adapting care routines, caregivers' limited repertoire and the timing of PCCS (Theme 4).

## 4 DISCUSSION

This study examined the acceptability and formal caregivers' experiences of PCCS. Quantitative and qualitative findings converged to affirm PCCS as a helpful, easy‐to‐apply resource that promotes caregivers' provision of person‐centred care. Our findings support the PCCS model,^34^ affirming the interplay between benefits for residents, caregivers, and their interpersonal relationship, and the symbiotic positive impact on caring environments and ease of care.

PCCS was perceived to enhance the mutual wellbeing of caregivers and residents, supporting happier, calmer, and more enjoyable caring experiences. Caregiver‐directed live music has been recognised to positively impact residents' emotional wellbeing and decrease symptoms of dementia.^24^, ^32^, ^52^ This supports a positive, caring environment, recognised as an essential component of person‐centred care.^10^, ^53^ Colleagues noticed the benefits of PCCS outside care routines, and caregivers utilised PCCS in other settings and activities, such as walking. This affirms the use of caregiver singing in all care situations.^26^ Our findings illustrate that residents' perceived improved mood and cognition supported better comprehension of verbal instructions, cooperation, and active participation in their own care. The latter occurs when people with dementia feel connected to self, others, and their environment.^54^ PCCS appeared to develop these meaningful connections by accessing the motivating nature of music^55^ and enhancing relational mutuality and reciprocity. Interestingly, quantitative findings suggested that caregivers were more deeply impacted by PCCS than the residents. Although this could simply indicate that caregivers were more certain of their experiences compared to their perceptions of the residents', it may suggest that PCCS promoted and recognised the caregiver's own connection with self and others as essential contributors to their wellbeing, capabilities, and capacities to offer person‐centred care. This reinforces the intrinsic value of caregivers' own personhood.^56^

Findings suggest that PCCS helped caregivers recognise, understand, and relate to residents' needs. Empathy and caregivers' positive emotional experiences seemed to foster self‐efficacy, reflected in their competence and confidence in applying PCCS and caring for residents. Greater self‐efficacy supports positive aspects of caregiving such as feelings of mutuality, personal growth and purpose, and job satisfaction^57^, ^58^ and reduces the burden of care.^59^ Caregivers' positive emotional experiences are vital in their provision of individualised care and personal relation to the resident.^60^ PCCS may support these positive aspects by increasing knowledge about dementia, enhancing communication, promoting attunement, and positively shifting caregivers' attitude. Their relationships seemed to move from task‐orientated, which diminishes personhood,^61^ to more aligned with the VIPS principles defined by Brooker^10^: PCCS helped caregivers adopt an individualised approach; better understand their perspective; and create a positive social environment. This contributes to current research on PCCS, live music training outcomes, music therapy skills‐sharing^34^, ^52^ and attunement in dementia care.^62^ Additionally, caregivers advocated for other care homes and caregivers to adopt PCCS, acknowledging it as useful and effective in various daily caring contexts. This is also noted in previous findings.^34^

Despite reservations about the effectiveness of person‐centred training programmes and the challenges of applying principles into practice,^42^, ^63^, ^64^ our findings suggest that PCCS may be a unique, concrete, and applicable vehicle for the delivery of person‐centred care in which practical music therapy skills encourages better attunement to residents and their needs. Our findings align with recognised outcomes of effective dementia training: a positive shift in caregivers' attitude towards dementia and people living with dementia,^65^ effective emotional regulation approaches for caregivers,^60^ and the creation of a caring, social environment.^53^

Some minor discrepancies were noted in the quantitative responses relating to ease of application of PCCS (Q13, Q19R) and whether the intervention works well (Q15, Q18R). These may be due to misreading of the negative statements. Additionally, despite the notion that PCCS created a positive shift in residents' mood, qualitative data intimated the unpredictable of residents' moods, acknowledging that outcomes relating to resident' mood are not always consistent.^66^ Additional challenges noted by caregivers included their limited song repertoire akin to previous PCCS findings.^34^ Despite this, findings illustrate the overall acceptability of PCCS training and caregivers' positive experiences of implementation.

### 4.1 Strengths and limitations

This study built on an initial study exploring PCCS, which, to our knowledge, is the first formal caregiver‐delivered, live music intervention training and protocol in South Africa. Results affirm the value of music therapy skills‐sharing. The inclusion of a large cohort of caregivers across several care homes supported PCCS' applicability and usefulness in care home settings irrespective of their resourced nature. Since the questionnaires were completed anonymously, we were unable to make comparisons caregivers' responses between care homes. The internal consistency was measured as high (Cronbach's *α* = .9), indicating that items were possibly similar and some items may have been redundant.

Likert scales pose some disadvantages in cross‐cultural research^67^ and responses may have been affected by language and cultural differences, and feelings of obligation. Also, writing feedback is potentially challenging as the majority of caregivers speak English as a second or third language and might not express themselves adequately in writing. This may suggest that findings be interpreted with some caution. Caregivers might have offered more verbal feedback through interviews which would have afforded more qualitative data. Priori assumptions possibly influenced the coding process, despite maintaining reflexivity through regular discussions. Although the management of two care homes attended the workshops, the study did not assess the impact of their involvement for the caregivers and their implementation of PCCS.

### 4.2 Recommendations

Translation of the workshop into two additional South African languages (Afrikaans and isiXhosa) and the inclusion of booster workshops could maximise comprehension and sustainability of PCCS in care homes. Further research is recommended: an intervention study would offer further insights into the implementation and sustainability of the PCCS training; expanding the studies to include residents in the early stages of dementia; a cost‐effectiveness study would offer insights into the financial feasibility of PCCS; and development and testing of the PCCS questionnaire for evaluation of general training in person‐centred care interventions.

## 5 CONCLUSION

This study aimed to investigate the acceptability of PCCS and experiences of caregivers' implementation thereof in the daily care of people with dementia in care homes. Findings suggest that PCCS is a helpful, easy‐to‐implement resource that contributes to the caregivers' delivery of person‐centred care by positively impacting residents' wellbeing, caregivers' emotional capacity, their empathic connection, the caring environment, and ease of care. By eliciting caregivers' own musicality and sharing music therapy techniques, caregivers are better equipped to recognise and attune to the residents' needs. PCCS may support the positive aspects of caregiving by shifting caregivers' understanding of dementia and promoting mutuality and reciprocity of relationship. Findings show a high level of acceptability of the PCCS protocol and support the notion of skills‐sharing by music therapists. This builds on previous findings^34^ and offers promising evidence of the acceptability and effectiveness of PCCS training. Residents did not always respond positively as attributed to their mood and potential disinterest in music. Further quantitative and qualitative research would benefit our understanding of caregiver‐delivered live music in relation to self‐efficacy, empathy, attunement and the impact on the caregiver's own personhood. It would be valuable to determine the training barriers in caregiver education in South Africa, whether implementation and positive impact of the PCCS training is maintained over time, especially relating to caregivers' attitudes, and whether it is a sustainable practice for caregivers in this context.

## AUTHOR CONTRIBUTIONS

**Karyn Stuart‐Röhm**: Conceptualisation; methodology; formal analysis; project administration; writing—original draft; writing—review and editing; visualisation. **Imogen N. Clark**: Conceptualisation; methodology; formal analysis; supervision; writing—review and editing; visualisation. **Felicity A. Baker**: Conceptualisation; methodology; formal analysis; supervision; writing—review and editing; visualisation. All authors approved the manuscript for submission.

## CONFLICT OF INTEREST STATEMENT

The authors declare no conflict of interest.

## ETHICS STATEMENT

Ethics approval was granted by the Human Research Ethics Committee of The University of Melbourne (Ethics ID: 22974) before recruitment.

## Supporting information

Supporting information.

## Data Availability

The data supporting the findings of this study are available within the article and from the corresponding author upon reasonable request.
