# Supplementary material for: Person‐centred caregiver singing for people living with dementia in South Africa: A mixed methods evaluation of acceptability, feasibility, and professional caregivers' experiences
Source: Health Expect. 2023 Nov 17;27(1):e13915. doi: 10.1111/hex.13915 (PMC10726267; doi:10.1111/hex.13915)
Supplement: Supplementary file 1 — Supporting information. [file HEX-27-e13915-s001.docx]

Appendix A: Questionnaire

**Person-Centered Caregiver Singing Questionnaire**

Please fill out this rating scale openly and thoroughly. Circle the numbers based on your experiences and opinions about the singing you have been doing with the residents on the dementia unit.

What is your gender? ⎕ Female ⎕ Male ⎕ Transgender ⎕ Intersex ⎕ other ⎕ prefer not to say

What is your ethnicity? ⎕ Coloured ⎕ isiXhosa ⎕ Caucasian ⎕ Zulu Other: __________________________________

What is your age? ________ (in years)

**PLEASE READ THE STATEMENTS CAREFULLY!**

| **Strongly**  **disagree** | Disagree | Neutral | Agree | **Strongly**  **Agree** |
| --- | --- | --- | --- | --- |
| 1 | 2 | 3 | 4 | 5 |

**Resident’s response to the Person-Centered Caregiver Singing**

***During the singing, the resident:***

1) Looks at me more than usual 1 2 3 4 5

2) shows a positive change in mood: smiling/laughing 1 2 3 4 5

3) Seems more at ease and comfortable 1 2 3 4 5

4) Seems to follow instructions better 1 2 3 4 5

5) Seems more talkative 1 2 3 4 5

6) Seems to be more compliant / cooperative 1 2 3 4 5

***Any other things you see in the resident when you do the person-centered caregiver singing?***

____________________________________________________________________________________________________________________________________________________________________

**My experience of the Person-Centered Caregiver Singing**

| **Strongly**  **disagree** | Disagree | Neutral | Agree | **Strongly**  **Agree** |
| --- | --- | --- | --- | --- |
| 1 | 2 | 3 | 4 | 5 |

***During the singing:***

7) I feel good/happier/content/lighter afterwards 1 2 3 4 5

8) I look at the resident more often 1 2 3 4 5

9) I enjoy using this way of singing 1 2 3 4 5

10) I give fewer verbal instructions 1 2 3 4 5

11) I connect better with the resident 1 2 3 4 5

12) I feel more confident when caring for residents 1 2 3 4 5

***Any other comments about how you feel when you do the person-centered caregiver singing?***

____________________________________________________________________________________________________________________________________________________________________

| **Strongly**  **disagree** | Disagree | Neutral | Agree | **Strongly**  **Agree** |
| --- | --- | --- | --- | --- |
| 1 | 2 | 3 | 4 | 5 |

**In general**

13) I found it easy to do Person-Centered Caregiver Singing 1 2 3 4 5

14) This has changed how I look at the person with dementia 1 2 3 4 5

15) I do not think this caregiver singing intervention works 1 2 3 4 5

16) This made helped make care routines easier 1 2 3 4 5

17) The workshop was easy to understand 1 2 3 4 5

18) I think singing works well with people with dementia 1 2 3 4 5

19) It was difficult to do person-centered caregiver singing 1 2 3 4 5

20) I will continue to do caregiver singing with residents 1 2 3 4 5

Any other comments you would like to make regarding your experience of the **Person-Centered Caregiver Singing?**

______________________________________________________________________________________________________________________________________________________________________________________________________________________________________________________

__________________________________________________________________________________

🎜 Thank you for your time 🎜
